# Cellular and molecular characterization of gametogenic progression in ex vivo cultured prepuberal mouse testes

**DOI:** 10.1186/s12958-017-0305-y

**Published:** 2017-10-18

**Authors:** J. Isoler-Alcaraz, D. Fernández-Pérez, E. Larriba, J. del Mazo

**Affiliations:** Department of Cellular and Molecular Biology, Centro de Investigaciones Biológicas (CIB-CSIC), 28040 Madrid, Spain

**Keywords:** Spermatogenesis, Ex vivo culture, Gametogenic progression, Testis, Meiosis, microRNA

## Abstract

**Background:**

Recently, an effective testis culture method using a gas-liquid interphase, capable of differentiate male germ cells from neonatal spermatogonia to spermatozoa has been developed. Nevertheless, this methodology needs deep analyses that allow future experimental approaches in basic, pathologic and/or reprotoxicologic studies. Because of this, we characterized at cellular and molecular levels the entire in vitro spermatogenic progression, in order to understand and evaluate the characteristics that define the spermatogenic process in ex vivo cultured testes compared to the in vivo development.

**Methods:**

Testicular explants of CD1 mice aged 6 and 10 days *post-partum* were respectively cultured during 55 and 89 days. Cytological and molecular approaches were performed, analyzing germ cell proportion at different time culture points, meiotic markers immunodetecting synaptonemal complex protein SYCP3 by immunocytochemistry and the relative expression of different marker genes along the differentiation process by Reverse Transcription - quantitative Polymerase Chain Reaction. In addition, microRNA and piwi-interactingRNA profiles were also evaluated by Next Generation Sequencing and bioinformatic approaches.

**Results:**

The method promoted and maintained the spermatogenic process during 89 days. At a cytological level we detected spermatogenic development delays of cultured explants compared to the natural in vivo process. The expression of different spermatogenic stages gene markers correlated with the proportion of different cell types detected in the cytological preparations.

**Conclusions:**

In vitro progression analysis of the different spermatogenic cell types, from both 6.5 dpp and 10.5 dpp testes explants, has revealed a relative delay in relation to in vivo process. The expression of the genes studied as biomarkers correlates with the cytologically and functional detected progression and differential expression identified in vivo. After a first analysis of deep sequencing data it has been observed that as long as cultures progress, the proportion of microRNAs declined respect to piwi-interactingRNAs levels that increased, showing a similar propensity than which happens in in vivo spermatogenesis. Our study allows to improve and potentially to control the ex vivo spermatogenesis development, opening new perspectives in the reproductive biology fields including male fertility.

**Electronic supplementary material:**

The online version of this article (10.1186/s12958-017-0305-y) contains supplementary material, which is available to authorized users.

## Background

Spermatogenesis is a complex cell differentiation process ending in male gamete generation and which entails three main phases: spermatogonial proliferation and renewal, meiosis and spermiogenesis [1]. During the first step, spermatogonial stem cells (SSCs) proliferate generating a stem cell pool or differentiating into spermatogonial cells. In mice, this occurs around the third postnatal day [2–4]. Spermatogonial cells go through different stages being the most representative type A spermatogonia, intermediate spermatogonia and type B spermatogonia. These last differentiate into primary spermatocytes which initiates the meiotic process [5] ending in generation of haploid cells (round spermatids) [3, 6]. Finally, in spermiogenesis, the round and elongated spermatids suffer deep morphological and physiological changes that include the replacement of histones by nuclear protamines, the elimination of nearly all cytoplasm, a nuclear condensation and the acrosome and flagellum formation that lead into spermatozoa genesis [3, 7, 8].

The spermatogenic process requires a regulated fine-tuning, which includes genetic, metabolic and hormonal regulations and even specific physiological conditions such as an optimal testes temperature control, lower to the body temperature in placental mammals [9, 10]. This germ line regulation is also mediated by the participation of testicular somatic cells as the Leydig cells, located in the intertubular regions, the myoid cells organizing the tubular wall and the Sertoli cells inside of the seminiferous tubules orchestrating the cell-cell interactions with the germ cells [2, 11, 12].

Defined gene expression patterns regulate the spermatogenic process both at a coding [13–16] and non-coding gene level. Currently, it is known that small non-coding RNA (sncRNA) participation is crucial as a post-transcriptional regulator. Amongst the sncRNA types, microRNAs (miRNAs) and Piwi-interacting RNAs (piRNAs) are being studied as regulatory elements essential in gamete differentiation and in pathologies leading to infertility [17–21].

The complexity of this terminal cell differentiation system plus its high regulation has complicated the development of in vitro models capable to reproduce the entire spermatogenic process [22–24]. Furthermore, cellular and molecular interactions caused by gonad architecture [25] should also be considered when trying to recreate this complex process.

Pioneer studies carried out last century in the decade of the 20’s tried to facilitate and develop ex vivo spermatogenesis systems [26]. Up to 1960–1970, most of the in vitro spermatogenesis experiments were performed using organ culture methods. In some of them, the spermatogenesis reached the meiotic phase but such cultures proved ineffective to generate spermatids [27, 28]. During the 80’s, in vitro spermatogenesis experiments replaced organ cultures by cell cultures, although these last never produced functional haploid cells [29]. Use in cultures of support cells, as for instance Sertoli cells, in order to promote the entire in vitro spermatogenic process marked a milestone event [30], even though proving impossible to reproduce. Over the last few years different culture conditions have been modified, including media components, temperatures, oxygen concentrations and cell architecture, geared towards obtaining better systems to reproduce the spermatogenic process [31]. Finally in the year 2011 the group of Ogawa developed a testis explant culture system capable of completely reproducing the spermatogenic process [32]. The system based on maintaining tissue architecture on a gelatinous support soaked with a defined culture medium has created enormous expectation. Authors achieved to generate spermatozoa that in microinsemination assays allowed to bring forth viable progeny [32, 33].

Although this last methodology looks like a notable advance to study spermatogenesis, the method ought to be improved in order to reproduce fully the ex vivo spermatogenic process in a similar fashion as occurs in vivo. This requires to undertake a comparative analysis of the ex vivo differentiation progress with the corresponding in vivo developmental stages. We present here a comparative deep analysis of the cellular and molecular dynamics operating during ex vivo spermatogenesis, extending the culture period from prepuberal mouse testis up to three months of continuous culture and testing two different culture medium supplements. This has been achieved by characterizing development and differentiation stages of the spermatogenic process after different days in culture and by assessing different gene expressions playing a key role in spermatogenesis. This may well improve the method allowing to identify associated molecular markers, including small non-coding RNA expression patterns, to further improve and implement normal and pathological spermatogenesis studies.

## Methods

### Animals

*Mus musculus* strain CD1 was used as model, supplied by the bioterium of the Centro de Investigaciones Biológicas-Consejo Superior de Investigaciones Científicas (CIB-CSIC) and bred under specific pathogen-free (SPF), temperature (22 ± 1 °C) and controlled humidity (50–55%) conditions. Animals were housed exposed to a 12 h light/dark regime and had ad libitum access to food and water. Animals were used at the age of 6.5 days *post-partum* (dpp) and 10.5 dpp to study the culture progression when meiosis was initiated (10.5 dpp animals) or before the meiotic onset (6.5 dpp animals). All animals aged 6.5 dpp and 10.5 dpp were sacrificed by decapitation. Adult mice were sacrificed by cervical dislocation and were used as study controls.

### Tissue culture

Tissue cultures were performed following the protocols described by Yokonishi et al. (2013) [34].

### Culture medium

The culture medium used was α-Minimum Essential Medium (α-MEM) (Gibco, Maryland, USA). 10.1 g of α-MEM powder were dissolved in 500 ml of Ultra-Pure Milli-Q® water to prepare α-MEM 2X stock. To 100 ml of this medium, 20 ml of KnockOut™ Serum Replacement (KSR) (Gibco), 5.2 ml of sodium bicarbonate (7% *w*/*v*) (Merck, Darmstadt Germany) and 2 ml of Penicillin-Streptomycin (10,000 u/ml Penicillin; 10,000 μg/ml Streptomycin) (Gibco) were added and then completed to 200 ml with Ultra-Pure Milli-Q® water. To evaluate the effectiveness of an alternative culture medium, other set of experiments was carried out adding 8 g of rich-lipid bovine serum albumin (AlbuMAX™ I) (Gibco) instead of KSR. All media were sterilized by filtration through a 0.22 μm membrane using Stericup® Millipore Express® PLUS (Merck Millipore, Madrid, Spain) and stored at 4 °C until use.

### Culture mounting

An aqueous sterile solution of 1.5% (*w*/*v*) agarose (Conda, Madrid, Spain) was solidified into sterile dishes (60 × 15 mm) covering 70% of their volume. Once solidified, the gel was cut into hexahedron shapes of about 13x13x7 mm in size with a sterile blade. These hexahedrons were soaked in fresh culture medium in a dish during at least 24 h in a 5% CO_2_ 95% air atmosphere at 34 °C to replace their initial inside water conformation with fresh medium.

### Culture method

**Fig. 1:**
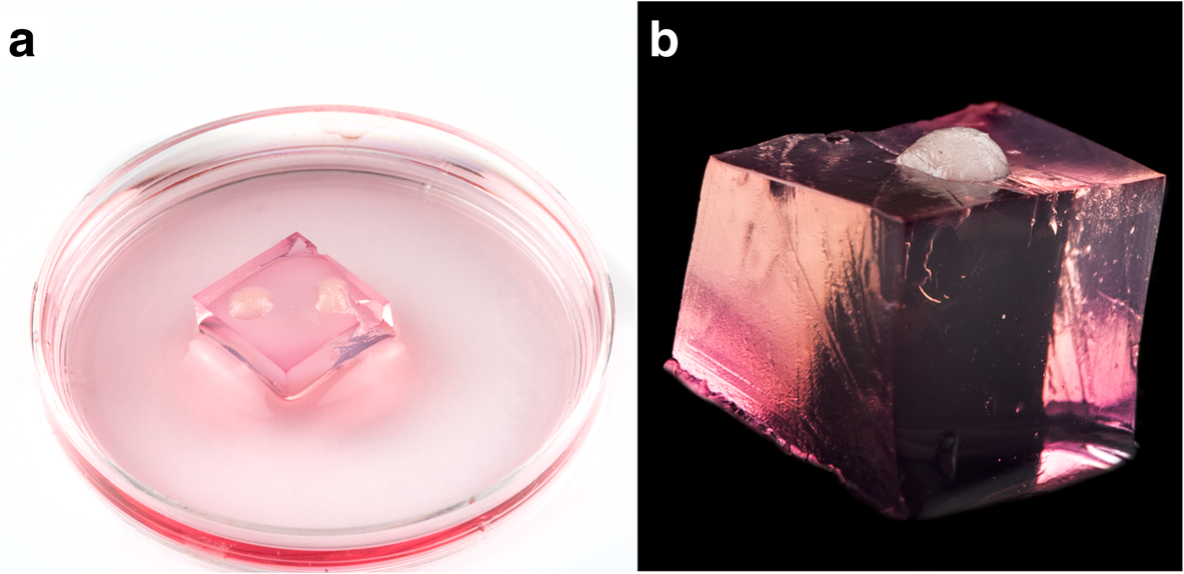
Real images of the culture method (**a**) 6.5 dpp testis explants cultured on an agarose hexahedron with KSR supplemented culture medium. (**b**) Characteristic disposition of a testis explant on an agarose block used for this culture method

The procedure described by [34] was followed. In brief, testes were removed from CD1 mice by *post-mortem* dissection taking care to eliminate the tunica albuginea in order to leave the seminiferous tubules in tight contact with the medium. Immediately, testes were placed at room temperature into dishes with fresh medium taking care not to disrupt the tissue structure. Subsequently, testes were cut into two halves to facilitate culture medium penetration. One to three hexahedrons were placed per well inside 6-well cell culture plates. Three testis explants were cultured on each hexahedron, one explant considered an entire testis in the case of the 6.5 dpp or half a testis in the case of the 10.5 dpp matter. Culture medium was added to each well up to 80% of the hexahedrons’ heights (Fig. 1). All procedures were carried out under sterile conditions and explants were cultured in a 5% CO_2_, 95% air atmosphere at 34 °C. Culture medium was changed twice a week.

### Cytological analysis of the spermatogenic culture progression

Three testis explants were removed after different specific days in culture and placed individually in a concave slide containing 100 μl of phosphate buffered saline (PBS). Cytological preparations were performed following classical procedures [35]. Tissue was dissociated in PBS using two fine forceps creating a monocelular suspension by continuous pipetting. The suspension was collected avoiding non-dissociated pieces and was centrifuged at 1000–1200 rpm at 4 °C for 15 min. The supernatant was removed and the cell pellet was resuspended adding a fixative solution (Carnoy solution): methanol (Merck) and glacial acetic acid (Merck) 3:1 up to a 5 ml volume. After fixation at 4 °C for at least 5 min samples were centrifuged using the same before mentioned conditions. The supernatant was removed and fresh fixative solution was added to the resuspended cells acting for 30 min at 4 °C. Thereafter, samples were once again centrifuged under the same conditions. The supernatant was removed, pellets were resuspended and fresh fixative solution was added depending on the required cell concentrations for microscope analysis (about 200–300 μl). For cellular smears, each cytological preparation was performed by spreading a single cell suspension drop over a glass slide with an inclination of about 45 degrees dropping from a height of about 25 cm. After drying, preparations were stained with UltraCruz® Mounting Medium with DAPI (Santa Cruz Biotechnology, California, USA) and analyzed under fluorescence microscopy (Olympus, Southend-on-Sea, UK). The different cell types identified according to nuclear morphology were recorded to establish the different germ cell proportions. Somatic cells such as Sertoli cells have not been considered to carry out the cellular profiling due to their lack of a clearly defined nuclear morphology during the early spermatogenetic development stages.

### Immunocytochemistry

Cytological preparations were performed by cytospin following standard procedures [36]. In short, 2–3 explants were removed from culture and dissociated in PBS using two fine forceps. The cell suspension was cytocentrifuged over round 12 mm diameter coverslips for 10–15 min at 1000 rpm. The cells attached to coverslips were fixed using 1% paraformaldehyde (PFA) [37] (Sigma-Aldrich, Spain) in PBS at 4 °C for at least 2 h. Alternatively, cells on coverslips were fixed in 100% methanol at −20 °C for 30 min to 1 h and post-fixed and permeabilized in acetone at −20 °C for 15 s followed by a 0.1% Triton X-100 in PBS rinse. Samples fixed in PFA were directly rinsed in 0.1% Triton X-100 in PBS for 10 min. Later, they were incubated with 5% bovine serum albumin (BSA) for 30 min followed by incubation with primary antibodies anti-SYCP3 mouse IgG_1_ (one commercial from Santa Cruz Biotechnology and the other kindly provided by Dr. J.L. Barbero), 2 μg/ml in a humidified chamber at 4 °C overnight. After washing cells three consecutive times for 10 min in PBS, they were incubated with the secondary antibody goat anti-mouse IgG_1_ Alexa Fluor® 488 (Invitrogen, USA) at 37 °C for 1 h in the dark. Afterwards, cells were rinsed 3 times in PBS and mounted with mounting medium containing DAPI (Santa Cruz Biotechnology). Cells were analyzed and recorded under fluorescence microscopy (Olympus) and laser confocal microscopy (Leica TCS SP5, Wetzlar, Germany).

### Total RNA isolation and RT-qPCR gene expression analysis

For eeach analysis, two testis explants were homogenized with a sterile mini Potter homogenizer after different culture times and RNA was isolated using TRIsure™ (Bioline, London, UK) and Direct-zol™ RNA MiniPrep commercial kit (Zymo Research, California, USA) according to the manufacturer’s specifications. Concentration and integrity of the isolated RNA was assessed making use of a Nanodrop™ ND-1000 (Thermo Fisher Scientific, Madrid, Spain) and Bioanalyzer 2100 (Agilent Technologies, Madrid, Spain). To analyze gene expression, RNA was reverse transcribed in the presence of 2.5 μM oligo d(T)_17_, 0.5 mM of each dNTP, 500 ng RNA and RNase-free water up to 13 μl, heated at 65 °C for 5 min followed by 1 min on ice, adding 1X SSIV buffer, 5 mM DTT, 2u RNAsin® Ribonuclease Inhibitor (Promega, Madrid Spain), 10u/μl SuperScript® IV Reverse Transcriptase (Invitrogen) and RNase-free water until obtaining a final volume of 20 μl. All reagents were incubated for 10 min at 52.5 °C. Reverse transcriptase was inactivated by incubating the mix at 80 °C for 10 min.

qPCR reactions were performed according to the MIQE guidelines [37] using for each reaction: 1X LightCycler® 480 SYBR Green I Master (Roche, Madrid, Spain), 0.0625 μM forward primer, 0.0625 μM reverse primer, 25 ng cDNA from the RT reaction and RNase-free water up to a volume of 10 μl. Primer sequences used are included in Additional File 1. qPCR conditions comprised 1 cycle at 95 °C for 3 min and 45 cycles of 15 s at 95 °C and 1 min at 60 °C.

To calibrate the primer efficiencies 5 serial dilutions were performed using adult testis cDNA. The analysis was carried out with 3 technical replicas and relative gene expression was calculated using the 2^-ΔΔCt^ method [38]. *Ppia*, *U6* and *H2afz* gene expressions were used to normalize data.

### Next generation sequencing (NGS) of small non-coding RNAs

RNA size separation, preparation of libraries and NGS were performed by a commercial agreement with the Beijing Genomics Institute (China) using 2 μg of total RNA isolated from each sample using the HiSeq 2000 (Illumina, California, USA). Small RNA-seq was performed at a depth of 10 million sequences and a 50 nucleotide extension. Thereafter used adaptors were trimmed and a read quality analysis using the program FastQC was assessed (http://www.bioinformatics.babraham.ac.uk/projects/fastqc/). Once the data had been filtered, reads were aligned against the mouse genome (mm10) using the Bowtie aligner [39]. Next, reads were sequentially aligned against different sncRNAs databases retaining the mapped reads: miRBase 21 [40], piRBase [#1]^1^, and Ensembl’s non-coding RNAs database [#2]^2^. Finally, reads that did not map against any of the sncRNAs databases nor against the mouse genome were classified as “unannotated”. The bioinformatic pipeline is included in the Additional File 2.

### miRNA RT-qPCR and NGS/RT-qPCR correlation

In order to validate NGS data, miRNA RT-qPCR was performed using custom stem-loop primers and TaqMan probes (Appled Biosystem) according to the manufacturer’s specifications. Three miRNAs selected at random among those highly expressed at 6dpp were selected: miR-let7a-5p, miR-99b-5p and miR-486a-5p. Their expressions were measured and compared to NGS data. For this, we performed reverse transcription using the same RNA used in NGS, following the manufacturer’s instructions by stem-loop primers (16 °C for 30 min, 42 °C for 30 min and 85 °C for 5 min). Next, the qPCR reactions were performed using TaqMan probes under the following conditions: 95 °C for 15 min, 45 cycles of 15 s at 95 °C and 60 s at 60 °C. The analysis was performed in 5 technical replicates and data were normalized using the ΔΔCt Livak method [38] and *U6* gene as reference. Each value both in NGS and RT-qPCR was considered relative to the value at 6dpp testis. Finally, data from qPCR and NGS were compared using Pearson Product Moment Correlation (*R* = 0.67; Additional File 3).

### Statistical analysis

Data were analyzed by a One-way ANOVA, followed by Dunnet’s test in order to compare each sample against an initial control situation and by Bonferroni’s test in order to compare couples of non-dependent samples. Data are expressed as mean (SD) and *p* < 0.05 was considered statistically significant. All statistical analysis was performed using the GraphPad Prism 5.03 software (California, USA).

## Results

### Cytological analysis of the spermatogenic progression in ex vivo cultures

Regarding the ex vivo spermatogenic progression analysis, testicular mouse explants of 6.5 dpp and 10.5 dpp were cultured and assessed at two extended culture periods (respectively after 55 and 89 days). Both cytological viability and spermatogenic progression were evaluated. All cultures were processed according to 2 different media supplements: KSR and AlbuMAX™ I, comparing their progression and cell differentiation stages.

An initial visual assessment of the morphological and external explant features evidenced that testis explants cultured on AlbuMAX™ media exhibited a reduced volume with respect to those explants cultured on KSR media. Moreover, the cytological analysis revealed that cells cultured on AlbuMAX™ media frequently presented an aberrant nuclear morphology, up to the extent that it was impossible to correctly characterize a cellular pattern and finally it was only possible to consider data obtained from the samples cultured on KSR media.

**Fig. 2:**
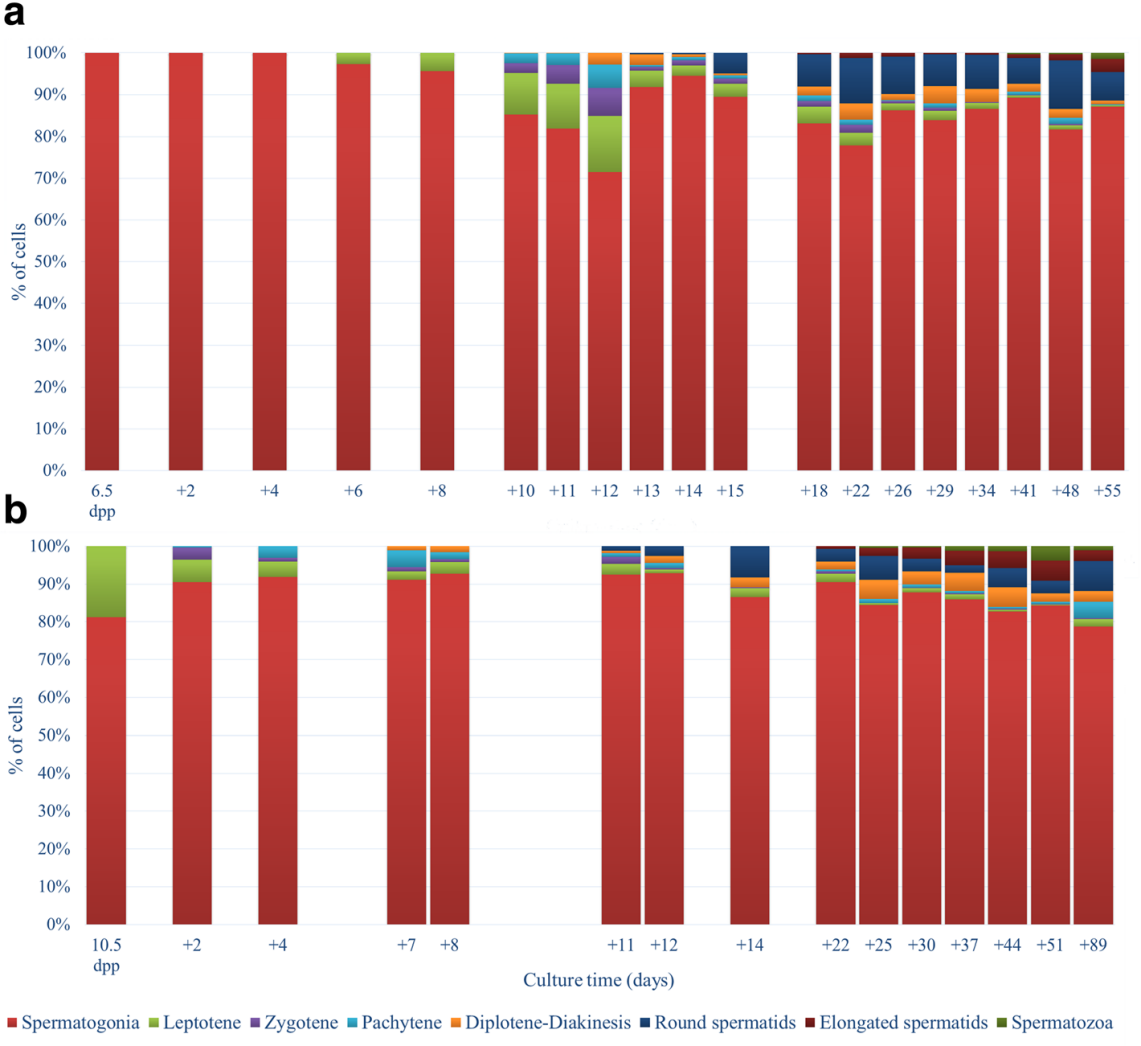
Spermatogenic progression at cellular level. Different germ cell type proportions along the culture period considering 6.5 dpp explants (**a**) and 10.5 dpp explants (**b**)

**Fig. 3:**
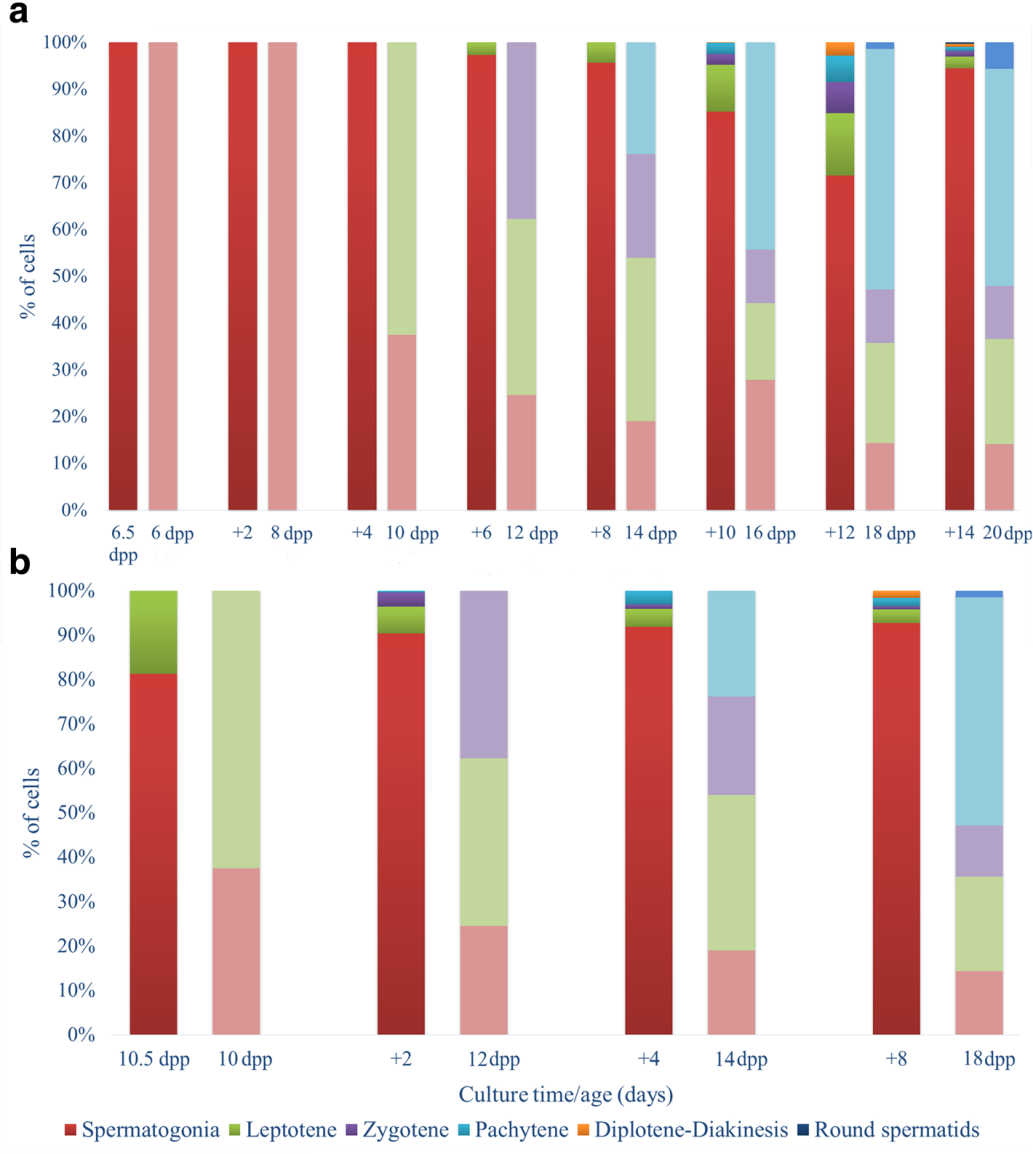
Spermatogenic ex vivo progression from 6.5 dpp testes (**a**) and 10.5 dpp testes (**b**) compared to the natural in vivo situation. Each column pair represents, to the left each sample’s different germ cell type proportion along the culture period. To the right is shown the in vivo germ cell proportion corresponding to the equivalent cultured explant age. The in vivo data are derived from Bellve et al., (1977)

Focusing on the culture results of the KSR supplemented media we have been able to confirm that according to the method’s initial authors [32], both 6.5 dpp and 10.5 dpp testes cultures have allowed a complete spermatogenic progression (Figs. 2A-B). The comparative culture differentiation analysis so far not performed, indicated that the progression from spermatogonia to elongated spermatids or even spermatozoa was suffering a relative delay compared with the classical normal established in vivo dynamics [3] (Figs. 3A-B).

**Fig. 4:**
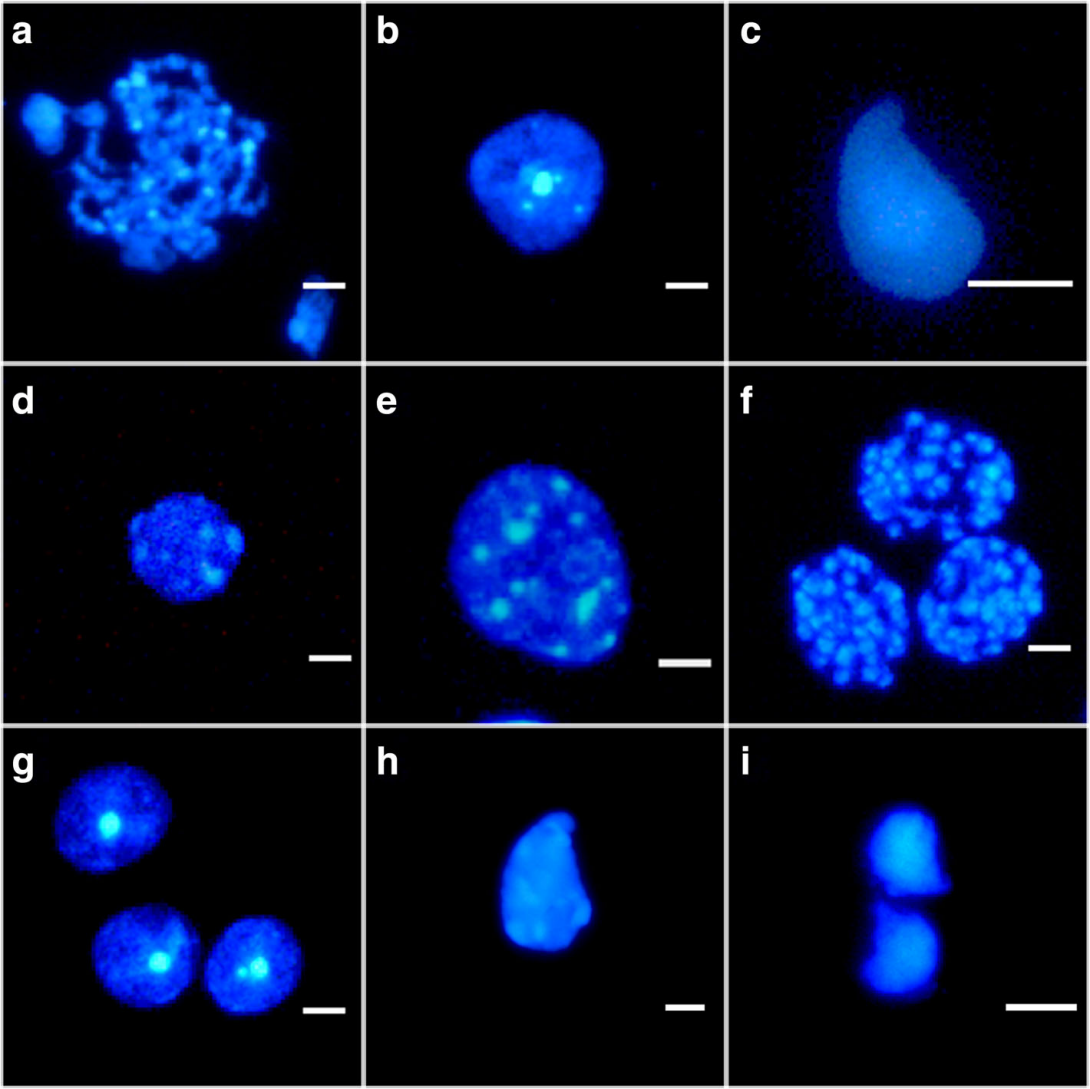
Nuclear morphology of the spermatogenic process in different cell types along the culture progression. (**a**) Pachytene cell at culture day 10 of a 6.5 dpp explant. (**b**) Round spermatid at culture day 13 of a 6.5 dpp explant. (**c**) Sperm head at the elongation phase after 41 culture days of a 6.5 dpp explant. (**d**) Type A spermatogonium at culture day 4 of 6.5 dpp explant. (**e**) Type B spermatogonium at culture day 6 of 6.5 dpp explant. (**f**) Spermatogonial mitosis at culture day 6 of a 6.5 dpp explant. (**g**) Round spermatids at culture day 11 of a 10.5 dpp explant. (**h**) Elongated spermatid at culture day 22 of a 10.5 dpp explant. (**i**) Sperm heads at culture day 25 of a 10.5 dpp explant. DAPI stained. Scale bars represent = 5 μm

As expected, in 6.5 dpp testes, meiosis was not initiated when cultures started. Nevertheless, after 6 days in culture a few cells initiated early meiotic prophase. After 10 days in culture the first pachytene cells could be identified (Fig. 4A); the in vivo equivalent to 16 dpp. However in vivo, about 50% of germ cells (without considering somatic cells) could be detected as pachytene spermatocytes, having appeared in this first wave of spermatogenesis about 3 days before [3]. At culture day 12 we detected the highest proportion of meiotic spermatocytes, although representing only about 30% of the germ cells. Remarkably, at 18 dpp more than 80% of germ cells can be identified as meiotic spermatocytes derived from in vivo testis samples, roughly corresponding to culture day 12 (6.5 dpp + 12). To specify within this developmental window better the differentiation progression dynamics we proceeded to assess the cellular profiles day by day commencing day 10 up to 15 days cultured. At culture day 13 the first round spermatids appeared even though in reduced proportion. Surprisingly, a similar low proportion of round spermatids was detected at the close equivalent of the 18 dpp in vivo period [3], notwithstanding that the ex vivo culture meiotic spermatocyte proportion was far lower than that observed in in vivo testis (Fig. 4B). This strongly suggests that the number of germ cells that exit meiosis and enter in a spermatid phase could depend on limiting factors regardless of the meiocyte population size. Finally, the first premature spermatozoa were detected at culture day 41 corresponding to 47–48 days in vivo testis, indicating a significant delay of more than 15 days [41] (Fig. 4C).

Throughout the cultures’ progressions a high spermatogonial cell proportion was detected, both of type A and of type B spermatogonia, although these last seemed to be more abundant (Fig. 4D-E). In any case, at the initial culture stages it was possible to frequently detect spermatogonial mitosis (Fig. 4F), suggesting that the spermatogonia type A proliferative activity had not been disrupted by the potential stress produced by culture.

In contrast to the 6.5 dpp explants, the 10.5 dpp explants corresponded to in vivo testes in which the meiotic phase had already begun. In these explants, the spermiogenic phase was detected after 11 culture days, time point allowing to observe the first round spermatids (Fig. 4G). After 22 days cultured the first elongated spermatid appeared (Fig. 4H) and further progressed into premature spermatozoa, observed since culture day 25 (Fig. 4I). In other words, the beginning of spermiogenesis was delayed (3 days) in contrast to the in vivo situation [3].

**Fig. 5:**
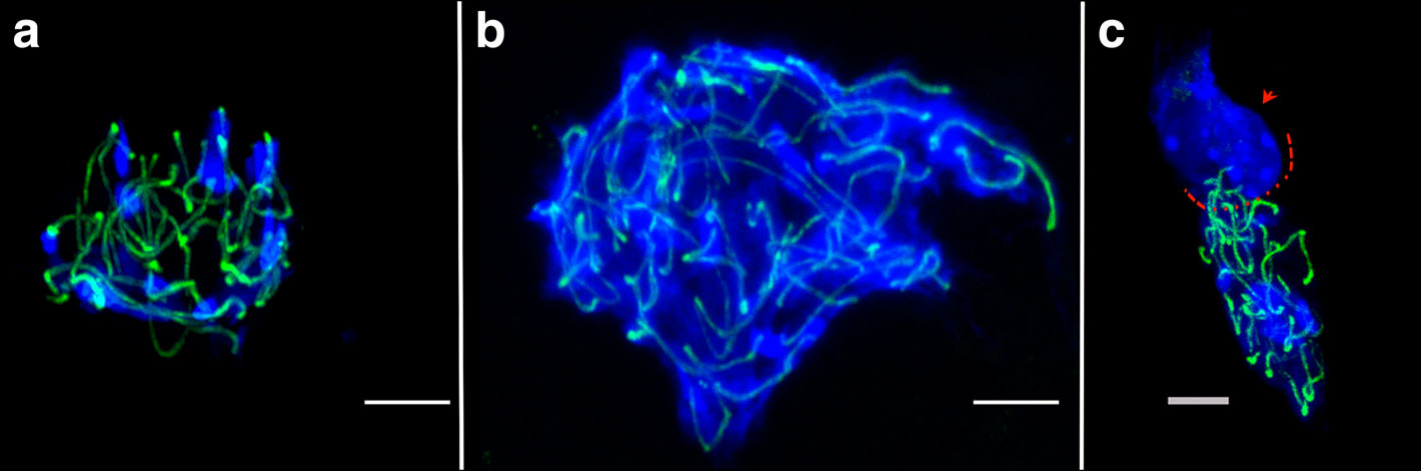
SYCP3 protein immunodetection in . In all samples the synaptonemal complex lateral elements are observed. The nuclei were stained with DAPI (in blue) and SYCP3 protein is shown in green. (**a**) Meiotic cell in pachytene phase after 15 days of culture of a 6.5 dpp explant. (**b**) Meiotic cell in late leptotene phase of an adult sample. (**c**) Two different cells (separated by dot lines in red) The upper cell (arrow) corresponds to a spermatogonia cell. The lower cell corresponds to a meiotic cell in pachytene phase after 14 culture days of a 6.5 dpp explant. Scale bars represent = 5 μm (**a**, **b**) and 25 μm (**c**)

To confirm the cytological progression, 3 different samples were analyzed by immunocytochemistry using antibodies against a meiotic process specific protein: SYCP3. This protein is considered a well-known pachytene marker being a component of the synaptonemal complex lateral elements. Explants of 6.5 dpp were processed after 14 and 15 culture days by cytospin procedures and immunodetection. Samples showed basic recognizable images of the synaptonemal complexes in agreement with the cytological analysis results (Fig. 5). In addition, similar results were also obtained by an alternative anti-SYCP3 antibody produced in our Center (J.L. Barbero) (data not shown).

### Differential gene expression analysis along the spermatogenic process

To validate at a molecular level the cytological culture progression and to assess the complete process, we performed RT-qPCR analyses of specific genes with defined expressions in in vivo spermatogenesis. For that purpose, RNA was extracted from 6.5 dpp explants after selected culture periods based on cytological results and processed by RT-qPCR. Gene expression patterns associated with germ cell differentiation and accompanying testis somatic cells were evaluated. Selected genes were: *Sycp1* and *Sycp3,* both highly expressed at the meiotic prophase encoding synaptonemal complex proteins, SYCP1 is localized in the central element and SYCP3 in the lateral elements [42]; *Prm2* and *Prm3* (both encoding protamine proteins) associated with histone replacement during spermiogenesis [43]; *Adam2* (encoding an endoprotease member of a disintegrin and metalloprotease family) related to postmeiotic testis expression [44]; *Trf* (encoding the transferrin protein) used as a Sertoli cell marker [45] and *Fn1* (encoding the fibronectin protein) handled as a seminiferous tubule myoid cell marker [45]. All data were normalized according to the relative gene expression levels at culture onset.

**Fig. 6:**
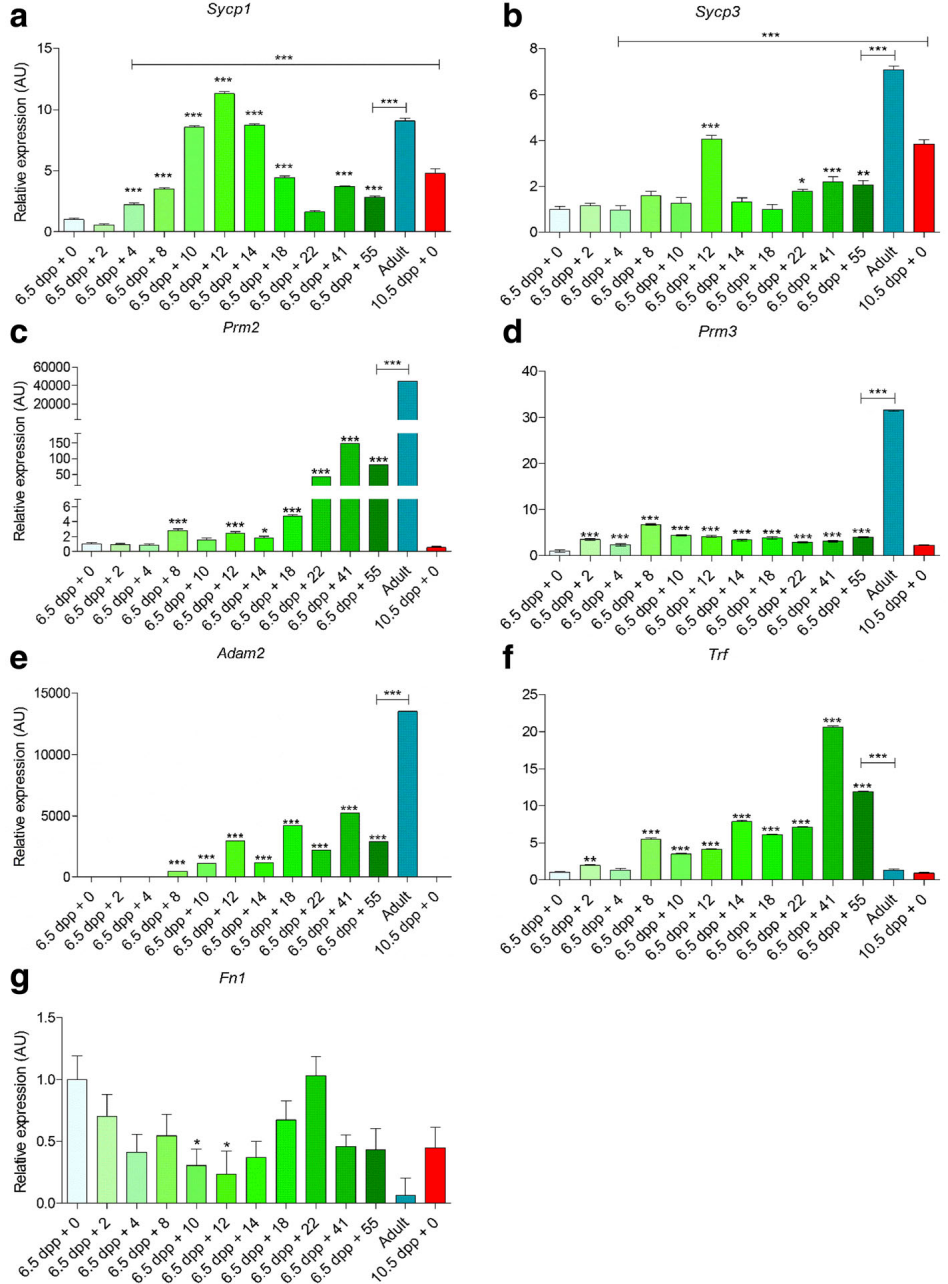
Relative gene expressions after RT-qPCR. Data corresponding to the cultured samples are shown in green, while data corresponding to adult testis samples and to 10.5 dpp explants are shown in blue and red respectively. Values are means (SD). All analyses were performed with 3 technical replicas. One-way ANOVA was applied in order to compare the cultured samples with the original situation (6.5 dpp + 0) using Dunnet’s post-test and to compare the last cultured sample to the adult sample and the hypothetically analogue sample using Bonferroni’s post-test. *, *P* < 0.05; **, *P* < 0.01; ***, *P* < 0.001

Along the culture spermatogenesis progression, the relative expression of *Sycp1* gradually rose until culture day 12, moment at which the expression was highest. The highest expression point of *Sycp1* fit with the highest meiotic cell proportion cytologically detected. Thereafter, the relative *Sycp1* expression gradually decreased until the culture day 22 subsequently stabilizing. In adult testis, this gene’s relative expression is high because a large proportion of cells are in meiotic prophase I. Finally, 10.5 dpp + 0 culture days evidenced a higher relative expression than that of its hypothetical time development analogue (6.5 dpp + 4 days of culture) (Fig. 6A). Regarding the relative expression of the *Sycp3* gene, a similar pattern to that manifested by *Sycp1* was detected nonetheless showing an abrupt expression increase at 6.5 dpp + 12 culture days. Again, *Sycp3* relative gene expression in adult testis was high, while expression at 10.5 dpp + 0 culture days was also higher than in its hypothetical equivalent of 6.5 dpp + 4 culture days (Fig. 6B). The expression patterns detected for the synaptonemal complex protein coding genes suggests that the in vitro spermatogenic progression cycles do not correspond to the in vivo detected pattern. In other words, it seems that the start of new spermatogenesis cycles in culture was obstructed by unknown reasons. This supposes an interesting challenge to undertake further investigations with respect to spermatogenesis regulation.

The *Prm2* gene encodes protamine 2, a protein involved in histone replacement in haploid cells during spermiogenesis. As depicted in Fig. 6C, as culture progressed, the relative accumulation of *Prm2* messenger RNA (mRNA) rose gradually. After culture day 22 expression levels increased significantly being associated with a spermatid and spermatozoa proportion increase. In adult testis, *Prm2* expression levels augmented substantially, reaching an expression approximately 50,000 times higher compared to the initial culture stages. The gene expression level of the 10.5 dpp + 0 culture days was quite similar to the analogue 6.5 dpp + 4 culture days corresponding to a postmeiotic cell absence. Unlike *Prm2*, *Prm3* expression encoding protamine 3 did not exhibit a gradual expression rise (Fig. 6D). Values after an unexplained low increase past 2 culture days, remained constant along all of the culture time with no evident differences among the hypothetical analogues (10.5 dpp + 0 culture days and 6.5 dpp + 4 culture days). In contrast, the gene expression in the adult testis was high. The *Prm3* and *Prm2* expression pattern differences could be due to dissimilar protamine 2 and 3 functions.

The *Adam2* gene encodes a metalloprotease holding an important role in oocyte and spermatozoa interactions. The relative gene expression levels gradually rose as culture time progressed (Fig. 6E). No gene expression was observed at the initial culture stages neither for 10.5 dpp nor for the hypothetical analogue 6.5dpp + 4 days. However, in adult testis the expression was higher compared to the other samples.

The *Trf* gene encodes transferrin, a Sertoli cell protein marker. The relative gene expression increased during culture progression (Fig. 6F), indicative that the relative Sertoli cell proportion and/or maturation increased during spermatogenesis progression. On the other hand, in adult testis the gene expression level was nearly zero, probably due to the scanty amount of Sertoli cells present in the sample compared to the abundant number of germ cells. Hypothetical analogues 10.5 dpp + 0 culture days and 6.5 dpp + 4 culture days revealed similar expression levels.

Last, the *Fn1* gene encodes fibronectin 1 protein, a myoid cell marker. The *Fn1* expression profile (Fig. 6G) was opposite to that observed for the *Sycp1* gene (Fig. 6A). As culture time progressed *Fn1* expression levels dropped all the way up to culture day 12, moment with the lowest levels. Beyond culture day 12 expression levels rose again up till day 22 and thereafter decreased again. This suggests that the *Fn1* mRNA proportion of the sample total RNA decreased comparatively with a boost of highly expressed meiotic genes along with an increase of differentiating spermatocytes, as also occurs in adults where the expression levels are very low as expected. The hypothetical analogues 10.5 dpp + 0 culture days and 6.5 dpp + 4 culture days expressed similar levels.

### Small RNA-seq analysis along the spermatogenic process

Small non-coding RNAs, mainly miRNAs and piRNAs comprise post-transcriptional regulators with important differentiation and development roles, also involved in the spermatogenic process [23, 46, 47]. In consequence and due to the lack of information about these regulatory molecules associated to in vitro or ex vivo gametogenic differentiation, we performed a sncRNA analysis focusing on miRNA expression. To understand how these small RNA molecules act in ex vivo cultures, sncRNA molecules were quantified by NGS approaches, initially assessing the 6.5 dpp and 10.5 dpp samples without undergoing culture, followed by evaluation of the 6.5 dpp and 10.5 dpp + 12 culture days on both media (KSR supplemented medium and AlbuMAX I supplemented medium) together with normal adult testes sample.

**Fig. 7:**
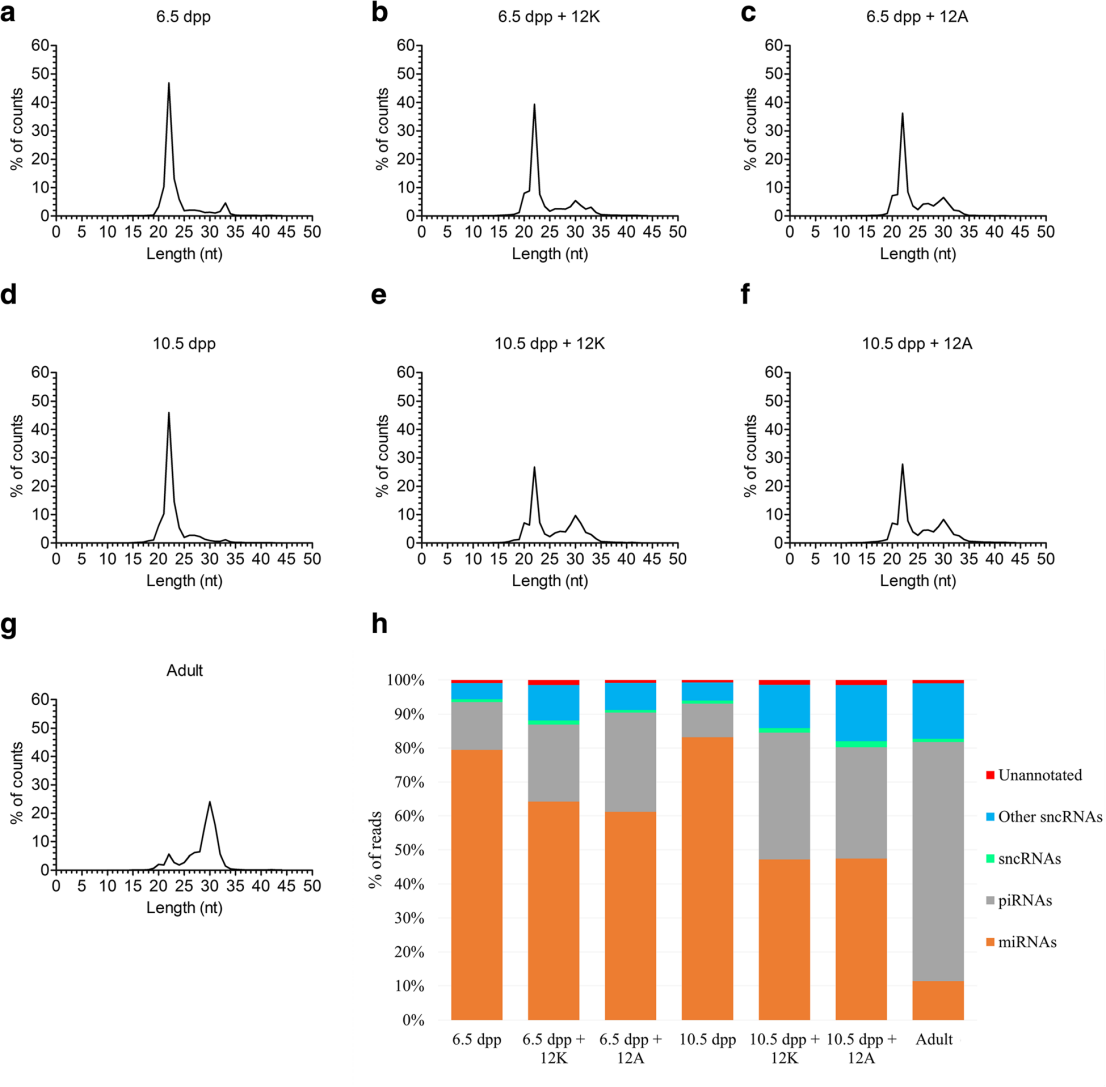
Length distributions of sncRNA reads subsequent to NGS. (**a**-**g**) Count proportions according to length are depicted for each sample. (**h**) The read proportions mapped against different databases: in orange (miRBase 21), in grey (piRBase), in green (Ensembl’s non-coding RNAs database) and in blue (other small RNAs sequences of the mouse genome). Sequences that did not map to any database were classified as unannotated and are displayed in red

Sequencing distribution results after filtering evidenced that at the initial spermatogenic process stages a high percentage of counts with 22 nucleotides in size (nearly 50%) were retrieved. This fraction is representative of miRNAs. Thereafter, along the culture time progressed we detected in all cases (6.5 dpp + 12 and 10.5 dpp + 12 on both media) that the proportion of the 22 nt sequences (putative miRNAs) progressively was reduced (to 40% in 6.5 dpp + 12 and to 30% in 10.5 dpp +12). Finally, in the adult sample the miRNA levels accounted for only 25% percent of the counts corresponding to the miRNA hallmark size. This indicates as expected that the spermatogenesis process is concomitant with a relative miRNA level decrease (Fig. 7A-G). In contrast, piRNAs (26–31 nt) at the beginning of spermatogenesis process yielded a low level of counts (less than 5% of the counts), as the spermatogenesis process proceeded the piRNAs proportion gradually increased up to 25% of the counts in adult sample (Fig. 7A-G). In this sense, the culture progression seemed to follow the in vivo cell differentiation pattern. Moreover, NGS data were aligned against different databases: mm10, premiRBASE 21, piRBASE and Ensembl’s ncRNAs database. Results revealed again that miRNA levels decreased with culture time progression, in contrast to piRNA that increased levels with culture progression (Fig. 7H). The relative piRNA increase appeared to respond to germ cell presence, which could be due to the piRNA main known function of germ cell protection against transposable elements [18, 48, 49].

Data confirmed that even at a postranscriptional gene regulation level mediated by sncRNAs, ex vivo culture progression patterns agreed with the cellular and the transcriptome levels.

**Fig. 8:**
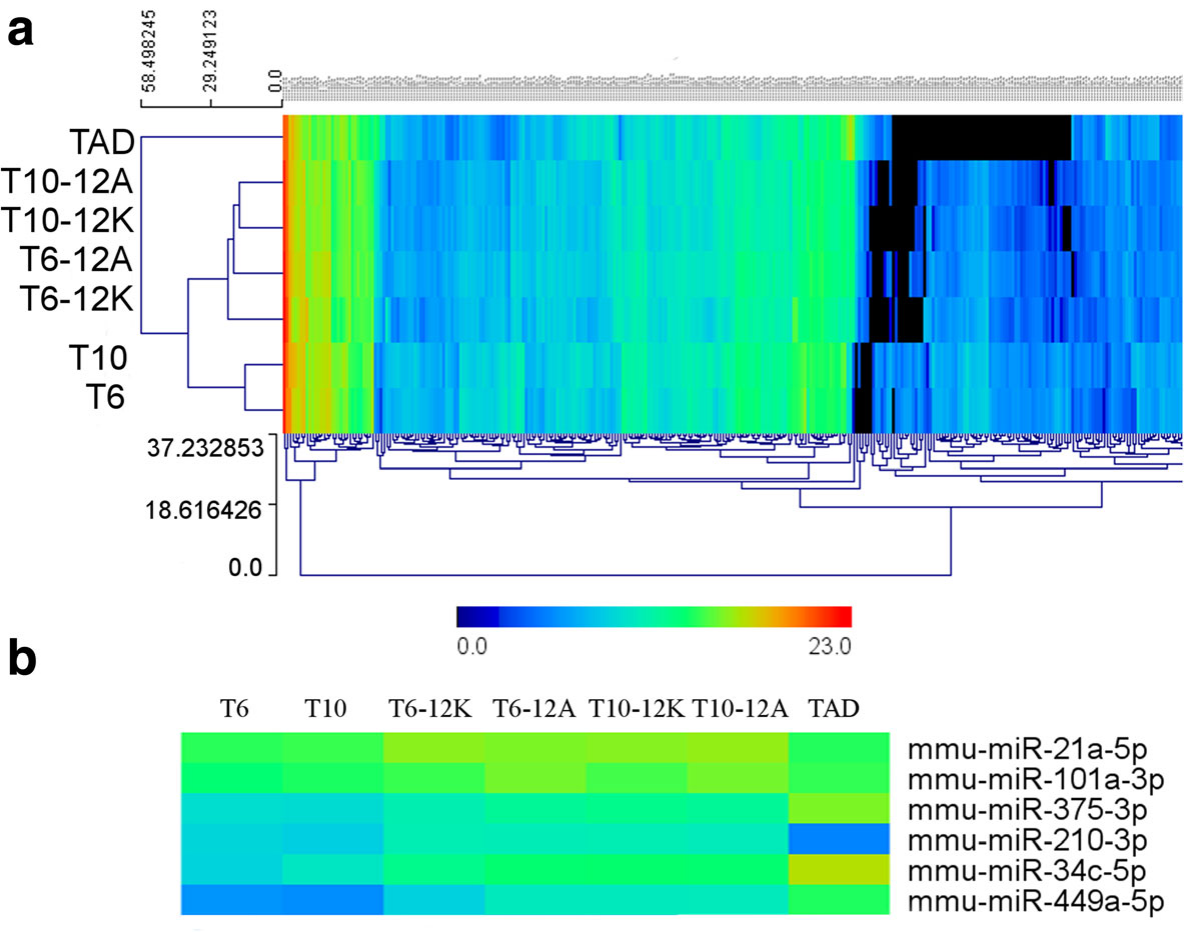
miRNA expression profiles. (**a**) Heat Map of all miRNAs after filtering and eliminating those with less than 50 reads. Hierarchical clustering analysis: T6 (6.5 dpp testis samples), T10 (10.5 dpp testis samples), T6-12 K (6.5 dpp testis cultured 12 days on KSR supplemented medium), T6-12A (6.5 dpp testis cultured 12 days on AlbuMAX I supplemented medium), T10-12 K (10.5 dpp testis cultured 12 days on KSR supplemented medium), T10-12A (10.5 dpp testis cultured 12 days on AlbuMAX I supplemented medium) and TAD (adult testis sample). Scale represents the relative miRNA expression levels adjusted to a logarithmic scale. (**b**) Heat Map of miRNAs with unusual expression patterns

A deeper data analysis revealed that a total of 331 different miRNAs were detected (discarding the low expressions corresponding to miRNAs with less than 50 reads). A miRNA hierarchical clustering analysis revealed 3 main signatures: one corresponding to the prepuberal testis before culture (T10 and T6), another at the other extreme corresponding to adult testis (TAD) and an intermediate gene expression signature pattern corresponding to the ex vivo culture progression (Fig. 8A). In general, as mentioned before, the expression of most miRNAs decreased with samples being more highly differentiated. Nevertheless, some miRNA exceptions increased their levels with samples being more highly differentiated (Fig. 8B), as for instance miR-34c-5p, miR-449a-5p or miR-375-3p. Remarkably, other miRNAs exhibited a higher expression in cultured cells such as: miR-101a-3p, miR-210-3p and miR-21a-5p, possibly indicative that culture conditions could directly affect miRNA expression in differentiating cells.

## Discussion

During nearly 100 years multiple attempts to implement suitable methods able to reproduce in vitro the spermatogenesis process had been unsuccessful. Recently, Saitou’s group published a promising method allowing male germ cells to be generated [50]. The system was basically based on the cell turn of embryonic stem cells into primordial germ cells, which once transplanted to male or female mice were capable of generating respectively fertile spermatozoa or oocytes. Nevertheless, results are nowadays being questioned [51] due to differences obtained among different studies. The ex vivo culture method developed by Sato et al. in 2011 [32] put to the test in this study could be considered less sophisticated than that carried out by Saitou’s group [50], although its simplicity could facilitate further implementation and development of new applications. Then again, the method requires a deep evaluation in order to bring about improvements allowing to be used further in research and to develop novel applications, this indeed having been the goal of the present study.

At a cytological level, we analyzed deeply the progressive cell type differentiation until developing into spermatozoa. The original study achieved in long time culture experiments to maintain cultures 70 days observing the last spermatozoa at day 60 [32]. This study has succeeded to induce and maintain the spermatogenesis process along a period of 3 months (89 days), even detecting spermatozoa after such a long period of time, although when RNA of these samples was purified the concentration was much lower compared to other samples purified in the early culture periods. Additionally, Sato et al. performed culture using different media and supplements, including those that we have used in our experiments. These authors, according to their results, considered that the addition of KSR as a medium supplement was essential to induce and maintain the spermatogenesis process in culture. They tested different elements of this supplement, describing that the lipid rich bovine serum albumin (AlbuMAX I) was the critical and indispensable factor of the medium. Thereafter Sato et al. used this element as a medium supplement instead of KSR and observed the same results. We have however not obtained the same results supplementing media with KSR compared to AlbuMAX I, so we believe that this last compound could be necessary but not sufficient in order to maintain ex vivo testis culture. Recently, Chapin et al. evaluated different culture conditions and media supplements trying to improve this method as a screen for spermatogenic toxicity. Although they finally ceased this attempt, they described melatonin as a culture medium supplement that improved germ cell production [52]. This is an important factor to consider for further studies.

In 1977, Bellve et al. characterized the comparative proportions of the different cell types present in the mouse seminiferous tubules throughout germ cell development and differentiation [3]. In the present study we have compared our ex vivo spermatogenesis development results to the in vivo data of Bellve. Comparing results whilst considering only germ cells, we noticed that ex vivo spermatogenesis presented a relative high proportion of spermatogonia in relation to the total number of cells. In cytological prepuberal animal preparations, we could not unquestionably identify Sertoli cells due to their nucleus primary morphology. Regarding to the *Trf* gene expression levels [45] the pattern was also inconclusive. This suggests that the ex vivo culture method could induce in Sertoli cells modifications that further merit analysis and improvement of the culture method in order to maintain the correct correlation between the Sertoli and germ cells as to achieve a normal spermatogenesis promoting proper fertility [53, 54]. It is also tempting to speculate with the idea that a precise and correct Sertoli maintenance of the spermatogenic cell structure and metabolic support [2] in this type of culture could facilitate successive spermatogenesis cycles. Another potential explanation for the low Sertoli cell proportion detected could be to have used KSR as a medium supplement. Recent studies performed with this compound have verified that KSR inhibits the germ cell capacity to enter into mitotic arrest thus deregulating the cell cycle control [55]. This could come about to be a reason why the spermatogonia population divided so much and accumulated. Moreover, it has been demonstrated that KSR affects the Sertoli cells by inhibiting their differentiation, which is very important at the postnatal stages [56], which could also explain why in the ex vivo culture these cells have problems to promote successive spermatogenetic cycles.

In addition to the fact that the spermatogonia cells were found in a high proportion, from our cytological level data of the spermatogenetic progression we can conclude that, in general, some delay exists considering the ex vivo spermatogenesis process with respect to the natural in vivo spermatogenesis. This delay was more pronounced in the samples cultured from testis explants of 6.5 dpp compared to those samples cultured from testis explants of 10.5 dpp. This most probably is due to samples cultured from testis explants of 6.5 dpp not having entered meiosis when culture was initiated and the transition from spermatogonia to spermatocyte could require an additional complex regulation. In any case, the in vitro spermatogenesis progression has clearly been confirmed by SYCP3 protein immunodetection assays in 6.5 dpp explants after 14 culture days.

The spermatogenic dynamics evaluated at a cytological level was also analyzed at different spermatogenesis stages via differential gene marker expression. The *Sycp1* and *Sycp3* genes encoding synaptonemal complex proteins [57], displayed an expression pattern during the first culture days fitting with that observed in the cytological preparations, detecting as spermatogenesis progressed a gradual gene expression increase. Nevertheless, in the later culture stages gene expression levels decreased in line with the relative meiotic cell proportion decrease detected in the cytological preparations. When compared to adult testis, data advocated that the ex vivo testis cultures under the present conditions did not reflect enough capacity to produce stable, additional and sequential spermatogenic cycles. This point should be considered in future studies aimed to improve and optimize the ex vivo testis culture method.

Examining the expression of genes *Prm2* and *Prm3*, encoding protamine proteins, we found that the first correlated both with our cytological progression data and with what could be expected from the in vivo spermatogenic situation. However, the constant expression of *Prm3* during all of the culture period could be due to the nature of this protein, which is not considered a real protamine [58], being implicated in spermatozoa motility but not in chromatin compaction during the last spermiogenesis stages. Spermatozoa motility has not been well defined yet in the spermatogenesis developmental method analyzed in this study, which supports the interest to perform a deeper study of its basic features using the ex vivo experimental approach with modifications.

Expression of *Adam2*, as it could have been expected due to a metalloprotease function in the late spermiogenesis stages, increased in level as the spermatogenic process progressed in culture, being highly expressed during the final stages, which correlated with the haploid cell proportion that we identified in the cytological preparations.

Noteworthy*, Trf* expression levels also increased as culture progressed, although cytologically we were unable to identify unquestionably Sertoli cells with their characteristic adult nuclear morphology [59]. Oddly enough, after 41 culture days the *Trf* expression remained very high, in contrast with the adult testis gene expression allowing to detect only minimum mRNA levels. This regulation event, so far not described neither in vitro nor in vivo, regarding the transferrin gene marker of Sertoli cells could be consequence of the Sertoli cell differentiation process under some potential stress conditions induced by culture. Considering that *Trf* has been identified as an androgen receptor–dependent gene [60], it will be interesting to further explore in future pathologies related to male infertility that reflect a similar *Trf* expression pattern.

Finally, Fn1 showed a curious relative expression pattern, being almost completely reverse to that presented by *Sycp1* and opposite to the highest meiotic cell proportion. Nevertheless, we have so far not found a logical explanation for this event, although it could be participating in similar adaptive mechanisms such as those suggested for the *Trf* gene.

Expression dynamics data of the two genes coding for two somatic cell structural proteins, evidenced that the entire physiological system was adapting to the culture conditions in order to facilitate cell differentiation. Moreover, the use of cellular and molecular markers as we have done in this study, will enable to optimize and expand the application and development of the spermatogenic culture system initiated recently by Sato’s group.

Deep sncRNA sequencing has allowed to attain an initial assessment of the miRNA and piRNA dynamics throughout ex vivo culture. We have verified that during the spermatogenesis progress miRNA expression decreases and piRNA expression increases. The piRNA level increase after 12 culture days correlates with the highest meiotic cell population extent observed in cytological preparations [48, 49]. However, some of the miRNAs identified in this study have not followed this expression pattern, for instance, miR-34c-5p and miR-449a-5p showed higher expressions with more differentiated cells, suggesting important roles at the final spermatogenic process stages, as has also been observed in previous studies [21, 61, 62]. The miR-34c-5p and miR-449a-5p dysregulations have been related with murine oligoasthenoteratozoospermia and sterility [63]. The highest miR-101a-3p expression level was detected in those samples cultured with the AlbuMAX I supplemented medium. As it has been observed in murine TM4 Sertoli cell cultures this miRNA was up-regulated when Sertoli cells were treated during 24 h with the endocrine disruptor nonylphenol [64], inducing Sertoli cell death by inhibiting testicular Ca^2+^ [65]. The fact that this miRNA was up-regulated in samples cultured with AlbuMAX I could explain why those samples showed a certain aberrant cell morphology thus entailing a potential marker. In our study miR-210-3p and miR-21a-5p manifested the highest expressions in all samples that were cultured on both media, in the same line miR-210-3p was up-regulated in patients with maturation arrest and hypospermatogenesis [66] and miR-21a was up-regulated promoting the self-renewal of mouse SSCs [67]. This could be the reason of the high spermatogonia proportion that we detected in all cultured samples, suggesting a problem with successive spermatogenesis waves as evidenced by the methodology used. Data suggest that all these miRNAs are closely implicated in the proper functioning of the spermatogenic process and could be prospective biomarkers using this ex vivo culture approach. Validation of NGS data were performed by RT-qPCR in tree miRNAs selected at random among the highly expressed at 6.5 dpp miRNAs. The correlation between NGS and RT-qPCR is positive and significant.

## Conclusions

The present work have validated by both cytological and molecular analyses of the complete differentiation process the methodology described by Sato’s group (2011) that allows bringing about in mice the entire spermatogenic process from prepuberal spermatogonia to spermatozoa. In spite of this, the ex vivo progression of both 6.5 dpp and 10.5 dpp testis explants revealed a relative delay in relation to the classical natural in vivo process together with difficulty of restarting successive spermatogenic cycles. On the other hand, the biomarker gene expressions correlated with the cytologically detected progressions and with the functions and differential gene expressions identified in vivo. In addition, ncRNA NGS data revealed that in general as cultures progressed miRNA levels decreased and piRNA levels increased, displaying a similar pattern to the natural in vivo spermatogenesis. Nevertheless, other miRNAs with unusual expression patterns were detected that could help to understand more profoundly how different culture variants influence enabling to improve the ex vivo spermatogenic process potentially useful to carry out further studies.

## Additional files

Additional file 1: Forward and reverse primer sequences used in RT-qPCR reactions. In blue are shown the sequence of those genes used to normalize the data. (XLSX 9 kb)

Additional File 2: Bioinformatic pipeline of the NGS data analysis. From the NGS filtered reads (red) a length distribution was made on one way (yellow). On the other way, those filtered reads were aligned sequentially against different databases (pink). The reads mapping against any database (plus those that did not map in the last data base) (green) were recovered for the diverse bioinformatic analyses. (TIFF 2402 kb)

Additional File 3: Validation of miRNA NGS data. Correlation showing relative expression values from the expression of 3 miRNAs (miR-let7a-5p, miR-99b-5p and miR-486a-5p) measured by NGS in all experimental stages analysed, related to the value at 6.5 dpp developmental testis (variable X, NGS data). The RTq-PCR data from the same miRNAs in each same stage also relative to 6.5 dpp data measure using custom stem-loop primers and TaqMan probes (Applied Biosystems, variable Y) were compared. The data in the RT-qPCR correspond to five replicates. Pearson correlation is indicated. (TIFF 88 kb)

## Abbreviations

AlbuMAX™ I: Rich-lipid bovine serum albumin
BSA: Bovine serum albumin
CIB-CSIC: Centro de Investigaciones Biológicas-Consejo Superior de Investigaciones Científicas
dpp: days *post-partum*
KSR: KnockOut™ Serum Replacement
miRNA: microRNA
mRNA: Messenger RNA
ncRNA: non-coding RNA
NGS: Next Generation Sequencing
PBS: Phosphate buffered saline
PFA: Paraformaldehyde
piRNA: Piwi-interacting RNA
RT-qPCR: Reverse Transcription – quantitative Polymerase Chain Reaction
sncRNAs: Small non-coding RNA
SPF: Specific pathogen-free
SSCs: Spermatogonial stem cells
α-MEM: α-Minimum Essential Medium

## Footnotes

1 The piRNA database - piRBase. http://regulatoryrna.org/database/piRNA/. Accessed June 2016.

2 Non-coding RNAs. http://www.ensembl.org/info/genome/genebuild/ncrna.html. Accessed June 2016.
