# Supplementary figures and images for: Cellular and molecular characterization of gametogenic progression in ex vivo cultured prepuberal mouse testes
Source: Reprod Biol Endocrinol. 2017 Oct 18;15:85. doi: 10.1186/s12958-017-0305-y (PMC5648490; doi:10.1186/s12958-017-0305-y)

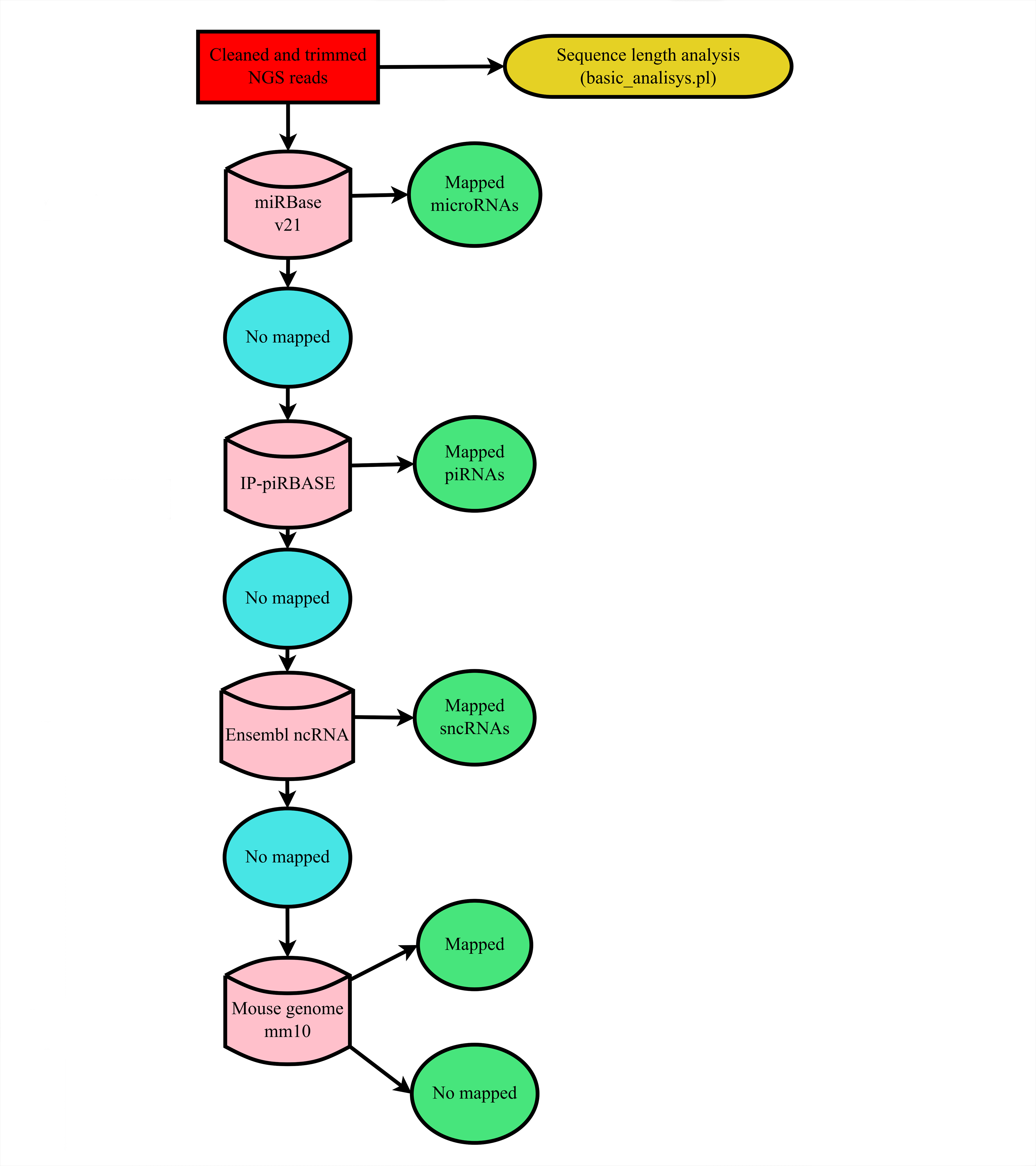

Supplement: Supplementary file 2 — Bioinformatic pipeline of the NGS data analysis. From the NGS filtered reads (red) a length distribution was made on one way (yellow). On the other way, those filtered reads were aligned sequentially against different databases (pink). The reads mapping against any database (plus those that did not map in the last data base) (green) were recovered for the diverse bioinformatic analyses. (TIFF 2402 kb) [file 12958_2017_305_MOESM2_ESM.tif]

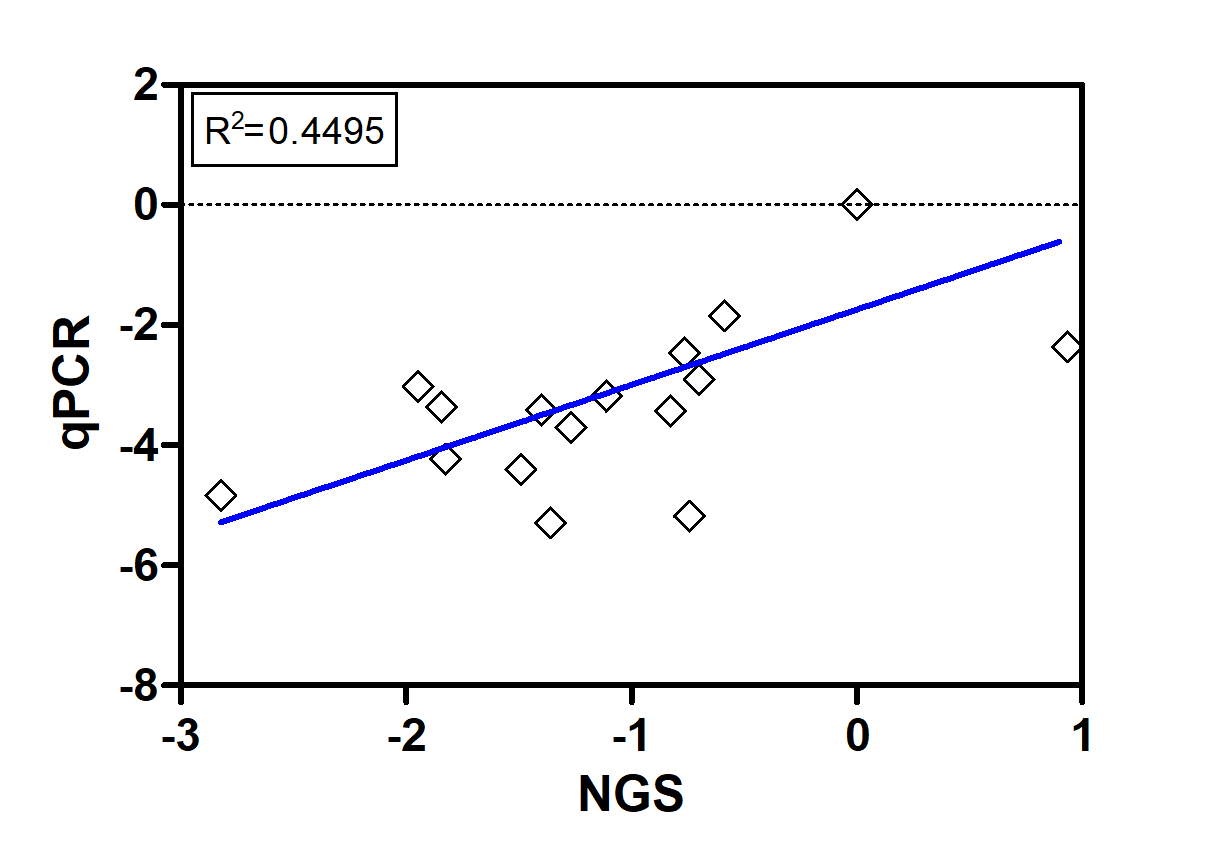

Supplement: Supplementary file 3 — Validation of miRNA NGS data. Correlation showing relative expression values from the expression of 3 miRNAs (miR-let7a-5p, miR-99b-5p and miR-486a-5p) measured by NGS in all experimental stages analysed, related to the value at 6.5 dpp developmental testis (variable X, NGS data). The RTq-PCR data from the same miRNAs in each same stage also relative to 6.5 dpp data measure using custom stem-loop primers and TaqMan probes (Applied Biosystems, variable Y) were compared. The data in the RT-qPCR correspond to five replicates. Pearson correlation is indicated. (TIFF 88 kb) [file 12958_2017_305_MOESM3_ESM.tif]
